# Beyond 532 and 1064 nm: The Role of 785 nm Ti:Sapphire Picosecond Lasers as a Complementary Platform in Tattoo and Pigment Treatment

**DOI:** 10.7759/cureus.113448

**Published:** 2026-07-27

**Authors:** Stefan Bigge

**Affiliations:** 1 Dermatology, Praxis Bigge Ungerechts, Cologne, DEU

**Keywords:** 785 nm, dermatologic laser, melasma, nd:yag, nevus of ota, picosecond laser, pigmented lesions, skin of color, tattoo removal, ti:sapphire

## Abstract

Picosecond lasers are increasingly used for tattoo removal and selected pigmentary disorders, but treatment outcomes remain dependent on wavelength, pulse duration, spot size, fluence, pigment chemistry, lesion depth, treatment endpoint, and skin phototype. This narrative review evaluates 785 nm treatment as an emerging complementary option alongside established 532 nm and 1064 nm platforms. A reproducible targeted PubMed/MEDLINE search and structured descriptive evidence extraction were used to identify direct clinical 785 nm studies, mechanistic work, comparator studies, and device-specific regulatory documents. The literature demonstrates that wavelength, laser source, pulse duration, and platform architecture are related but non-interchangeable concepts. Direct 785 nm evidence is limited to small prospective studies, pilot studies, retrospective series, and case reports. The most consistent clinical signal concerns selected cool-colored tattoo pigments, while reports in ephelides, benign facial pigmentation, melasma, and brown dermal melanocytosis remain preliminary. Experimental melanosome-disruption data provide mechanistic plausibility but do not define clinical treatment or safety thresholds. Device terminology also requires caution because nominal 785 nm outputs may be described differently across publications and technical documents. Overall, 785 nm should be considered a target-specific adjunct rather than a universal replacement for 532 nm or 1064 nm treatment. Larger comparative studies with standardized parameters, objective outcomes, longer follow-up, and broader representation of darker skin phototypes are required.

## Introduction and background

Laser treatment of tattoos and pigmentary disorders is based on the selective interaction between optical energy and target chromophores, principally tattoo particles and melanin-containing melanosomes, with competing absorption by endogenous chromophores such as hemoglobin. Wavelength determines the relative absorption profile and tissue penetration, whereas pulse duration, beam diameter, fluence, beam profile, cooling, treatment endpoint, and treatment interval influence the balance between pigment disruption and collateral tissue injury. Selective photothermolysis describes spatially confined injury when the wavelength, exposure time, and energy density are matched to a target [1,2].

Several technical terms require distinction. The laser source, or gain medium, is the material in which optical amplification occurs; titanium-doped sapphire, commonly abbreviated as Ti:Sapphire, is a tunable solid-state medium that can generate near-infrared wavelengths including 730 nm and 785 nm. A device platform may combine several sources, nonlinear frequency conversion, and interchangeable handpieces; therefore, a nominal wavelength does not, by itself, identify the gain medium or pulse duration. Stress relaxation time describes the approximate time over which mechanically induced pressure dissipates from a target, whereas thermal relaxation time describes heat dissipation. Picosecond pulses can approach the stress-confinement range of small pigment particles, increasing photomechanical fragmentation; however,​​​​​​​ pulse duration alone does not guarantee better clinical clearance [3-6].

Threshold fluence is the experimentally observed radiant exposure at which a defined physical change first occurs under specified conditions. A melanosome-disruption threshold measured in an ex vivo suspension is therefore not equivalent to a clinical treatment fluence or safety threshold. Melanosomes are endogenous organelles with relatively reproducible optical properties, whereas tattoo particles vary in composition, aggregation, size, depth, and encapsulation. Findings from melanosome models can support biological plausibility but cannot be directly extrapolated to human skin or tattoo-particle fragmentation.

The 532 nm and 1064 nm wavelengths ​​​​​​​remain foundational components of many tattoo and pigment platforms. The 1064 nm Nd:YAG wavelength has lower epidermal melanin absorption and greater penetration than shorter visible wavelengths and remains central for treating​​​​ black and deeper dark tattoo pigments. The 532 nm wavelength is frequently used for selected superficial warm-color pigments and epidermal pigmentation but interacts more strongly with epidermal melanin and hemoglobin. The 785 nm wavelength warrants separate appraisal from the neighboring 730 nm and 755 nm wavelengths because absorption, scattering, pulse duration, available spot size, and platform engineering differ sufficiently to prevent direct pooling. The objective of this review is to identify the direct clinical evidence for 785 nm, distinguish Ti:Sapphire-specific evidence from nominal wavelength labels, evaluate mechanistic and contextual evidence without over-extrapolation, and define a clinically conservative complementary role for 785 nm treatment.

## Review

Review methodology and study selection

This is a structured narrative review with a practical treatment-selection framework. PubMed/MEDLINE was the sole bibliographic database used for the primary literature search. The original searches were conducted on June 24-25, 2026, and repeated during the revision on July 15, 2026. The US Food and Drug Administration (FDA) 510(k) database was searched separately for device taxonomy and regulatory status. Backward citation checking and exact-title searches were used to verify key reports and bibliographic details.

The 24 predefined PubMed searches returned 743 query-level records before cross-query deduplication. After title-level deduplication and relevance screening, 65 unique records were retained in the screening log. Forty-three full-text articles or complete regulatory reports were assessed. Twenty-eight sources were included in the final synthesis: seven direct clinical 785 nm reports, one direct mechanistic study, two regulatory documents, and 18 comparator, contextual, or safety sources. Fifteen full-text candidates were excluded because they addressed noncutaneous applications, rejuvenation without a relevant pigment endpoint, combination treatment without an isolatable 785 nm contribution, duplicate cohorts, or insufficient device-specific information.

The eligibility criteria included peer-reviewed human studies reporting clinical use of 785 nm for tattoo or pigment targets; mechanistic studies directly evaluating wavelength-dependent pigment or melanosome disruption; comparative studies of picosecond versus nanosecond or Q-switched treatment relevant to interpretation; studies of 730 nm or 755 nm used only as neighboring-wavelength context; and official regulatory documents used only for device architecture and jurisdiction-specific indications. Searches were not restricted by publication year. English-language full text was required for detailed data extraction. Reports unrelated to cutaneous pigment, nonclinical engineering papers without a dermatologic target, and studies in which the 785 nm effect could not be separated from a multimodal intervention were excluded from the direct-evidence synthesis.

The single author performed screening and extraction in two stages: title and abstract screening followed by full-text eligibility review. For direct clinical studies, a structured descriptive extraction form was used to capture the study design, sample size, Fitzpatrick skin phototype, indication, device and stated laser source, pulse duration, fluence, spot size, number of passes, treatment sessions, interval, clinical endpoint, outcome instrument, evaluator blinding, follow-up, adverse events, funding, conflicts of interest, and study limitations. The term “appraisal” in the earlier version referred to this structured descriptive extraction and critical interpretation. No validated risk-of-bias instrument, GRADE (Grading of Recommendations Assessment, Development, and Evaluation) assessment, or formal certainty rating was applied; this is acknowledged as a limitation. No meta-analysis was attempted because the direct evidence consisted of heterogeneous case reports, single-arm studies, pilot studies, a retrospective series, and an experimental model with noncomparable outcomes.

Complete PubMed Search Strategy

A1 (n=11): ("785 nm" OR "785-nm") AND picosecond AND ("Ti:Sapphire" OR "titanium sapphire")

A2 (n=15): ("785 nm" OR "785-nm") AND picosecond

A3 (n=10): ("785 nm" OR "785-nm") AND picosecond AND (dermatology OR skin OR cutaneous)

B4 (n=2): ("785 nm" OR "785-nm") AND picosecond AND tattoo

B5 (n=1): ("785 nm" OR "785-nm") AND tattoo AND (blue OR green OR violet OR purple)

B6 (n=5): ("Ti:Sapphire" OR "titanium sapphire") AND tattoo

C7 (n=9): ("785 nm" OR "785-nm") AND picosecond AND (pigment OR pigmented OR pigmentation OR melanosis)

C8 (n=4): ("785 nm" OR "785-nm") AND ("benign pigmented lesion" OR lentigines OR freckles OR ephelides)

C9 (n=2): ("785 nm" OR "785-nm") AND melasma

C10 (n=2): ("785 nm" OR "785-nm") AND ("nevus of Ota" OR melanocytosis)

D11 (n=49): "532 nm" AND "1064 nm" AND picosecond

D12 (n=10): "532 nm" AND "1064 nm" AND picosecond AND tattoo

D13 (n=26): "1064 nm" AND picosecond AND tattoo

D14 (n=22): "532 nm" AND picosecond AND tattoo

E15 (n=63): picosecond AND (nanosecond OR "Q-switched") AND tattoo

E16 (n=15): picosecond AND ("Q-switched Nd:YAG" OR "nanosecond Nd:YAG") AND (tattoo OR pigment)

E17 (n=135): (("532 nm" OR "1064 nm") AND picosecond) AND (nanosecond OR "Q-switched")

F18 (n=68): picosecond AND (melanosome OR melanin)

F19 (n=51): picosecond AND wavelength AND (melanosome OR melanin OR pigment)

F20 (n=90): ("532 nm" OR "730 nm" OR "755 nm" OR "785 nm" OR "1064 nm") AND picosecond AND (melanosome OR melanin OR pigment)

G21 (n=24): "755 nm" AND picosecond AND tattoo

G22 (n=70): "755 nm" AND picosecond AND (pigment OR melasma)

H23 (n=2): "730 nm" AND picosecond AND tattoo

H24 (n=57): ("730 nm" OR "785 nm") AND picosecond

Picosecond Versus Nanosecond Treatment Is Not a Simple Hierarchy

A central interpretive pitfall is the assumption that a shorter pulse duration alone guarantees superior clearance. In a randomized controlled split-tattoo trial, Pinto et al. found no superior clearance with a 1064 nm picosecond Nd:YAG laser compared with a 1064 nm nanosecond Nd:YAG laser after two treatments. However, pain was lower with picosecond treatment [7]. Other prospective comparisons likewise demonstrate device- and indication-dependent, rather than universal, advantages [8,9].

For multicolored tattoos, Kono et al. demonstrated wavelength-specific performance within 532/1064 nm systems. The 1064 nm picosecond wavelength performed best for black pigment, whereas 532 nm picosecond treatment performed better for selected red and green components; post-inflammatory hyperpigmentation (PIH) and paradoxical darkening still occurred [10]. A later meta-analysis found broadly comparable efficacy for endogenous hyperpigmentation, a borderline trend favoring picosecond devices for exogenous tattoo pigment, lower pain, and lower rates of selected pigmentary adverse events; however, the pooled evidence was heterogeneous and not specific to 785 nm [11]. These findings support target-specific wavelength selection rather than a simple picosecond-versus-nanosecond hierarchy.

Device Taxonomy and Regulatory Status

Precise nomenclature is essential because wavelength, gain medium, pulse duration, and device platform are distinct variables. The cited FDA summaries describe HELIOS 785 Pico as a combined platform containing Q-switched 1064 nm Nd:YAG and frequency-doubled 532 nm KTP/Nd:YAG outputs with pulse durations of 5-10 ns, together with a gain-switched 785 nm Ti:Sapphire output with an approximately 600 ps pulse duration [12,13]. Consequently, the 532 nm and 1064 nm components of this platform should not be described as picosecond outputs.

The same documents list a US regulatory indication for the 785 nm channel limited to the removal of green and blue tattoos in Fitzpatrick skin types (FSTs) II-IV [12,13]. This statement is device- and jurisdiction-specific. Regulatory clearance establishes that a device met the applicable regulatory pathway for a stated indication; it does not constitute evidence of comparative efficacy, durable clearance, or safety across unlisted skin phototypes and should not be used as a substitute for clinical studies.

PicoWay is a separate configurable picosecond platform; its 730 nm data remain contextual. The study by Hong et al. [18] calls HELIOS IV a 785 nm picosecond Nd:YAG device, whereas HELIOS technical documents identify an Nd:YAG-based platform with a Ti:Sapphire 785 nm channel. Therefore, the report is labeled HELIOS IV 785 platform evidence without assigning a generic gain-medium label.

Direct 785 nm Clinical Evidence

Seven direct clinical reports were identified [14-20]. Tables 1-3 summarize their study design and platform characteristics, treatment parameters and outcome methods, and principal findings and limitations. Each table presents the individual variables separately to preserve device-specific interpretation and avoid combining nonequivalent data.

**Table 1:** Design and device characteristics of direct clinical 785 nm study components FST: Fitzpatrick skin type; NR: Not reported; ps: Picoseconds.

Study	Indication	Design	Participants	Target count	FST	Device/platform	Pulse duration
Bernstein et al. [14]	Multicolor tattoos	Prospective single-arm	15	22 tattoos	II-IV	PicoWay prototype Ti:Sapphire	300 ps
Chung et al. [15]	Ephelides	Case series	2	NR	NR	785 nm picosecond device; source NR	NR
Loh and Wu [16]	Nevus of Ota	785 nm case report	1	1 lesion	IV	PicoWay Ti:Sapphire	300 ps
Hong et al. [17]	Facial pigment disorders	Case series	3	NR	III-IV	HELIOS IV Ti:Sapphire	600 ps
Hong et al. [18]	Facial benign pigment	Prospective pilot	15	NR	II-IV	HELIOS IV 785 platform	600 ps
Choi et al. [19]	Melasma	Prospective single-group	18	NR	NR	HELIOS IV Ti:Sapphire	600 ps
Swali et al. [20]	Brown nevus of Ota	Retrospective series	17	NR	II-V	PicoWay Ti:Sapphire	NR

**Table 2:** Treatment parameters and outcome-assessment methods in direct clinical 785 nm studies DOE: Diffractive optical element; NR: Not reported.

Study	Protocol component	Fluence	Spot size	Sessions	Interval	Clinical endpoint	Outcome instrument	Blinding	Follow-up
Bernstein et al. [14]	Standard handpiece	1.1-3.1 J/cm^2^	2-4 mm	4	6-10 weeks	Immediate whitening	Cross-polarized photography; 11-point scale	Yes	8 weeks
Chung et al. [15]	Standard handpiece	1.2-1.3 J/cm^2^	3 mm	1	Not applicable	Slight darkening	Clinical photography	No	NR
Loh and Wu [16]	785 nm case	1.0 J/cm^2^	4 mm	3	1 month	Gentle frosting or erythema	Clinical photography	No	1-3 months
Hong et al. [17]	DOE mode	0.8 J/cm^2^	5 x 5 mm	3-4	4 weeks	Mild erythema or edema	Clinical photography	No	6 weeks
Hong et al. [17]	Focal mode	1.2 J/cm^2^	3 mm	3-4	4 weeks	Mild erythema or edema	Clinical photography	No	6 weeks
Hong et al. [18]	DOE mode	0.8 J/cm^2^	5 x 5 mm	5	1 week	NR	Clinical score; pigment indices	NR	Week 8
Hong et al. [18]	Collimated mode	0.3-0.4 J/cm^2^	7 x 7 mm	5	1 week	NR	Clinical score; pigment indices	NR	Week 8
Choi et al. [19]	DOE mode	0.4-0.6 J/cm^2^	5 x 5 mm	10	2 weeks	Mild erythema	Investigator score; Mexameter	NR	Week 22
Swali et al. [20]	Standard handpiece	1.0 J/cm^2^	4 mm	Mean 3.2	2-3 months	Dermal edema	Five-point visual scale	NR	NR

**Table 3:** Outcomes, adverse events, funding, and principal limitations of direct clinical 785 nm studies Follow-up refers to the period after the final treatment, when specified. The parameters are not interchangeable across devices. Evidence labels describe the study design only and do not imply formal GRADE certainty or long-term safety. NR: Not reported; PIH: Post-inflammatory hyperpigmentation; GRADE: Grading of Recommendations Assessment, Development, and Evaluation.

Study	Principal outcome	Acute adverse events	Pigmentary or textural events	Funding	Conflict of interest	Evidence design	Principal limitation
Bernstein et al. [14]	Highest mean clearance in purple, blue, and green components	Erythema; edema; one pinpoint bleed	No scarring or dyspigmentation reported	Manufacturer supported	Two manufacturer-employed authors	Prospective noncomparative	Small sample; short follow-up; no active comparator
Chung et al. [15]	Ephelides improved after one session	None reported	None reported	NR	NR	Case series	Two patients; subjective assessment; follow-up NR
Loh and Wu [16]	Greater than 75% clearance in both reported cases	None reported	No pigmentary alteration reported	No funding reported	External industry relationship	Case report	Only one 785 nm case; short follow-up
Hong et al. [17]	Visible improvement in mixed facial pigment disorders	None reported	No PIH or depigmentation reported	No funding reported	No conflict reported	Case series	Three patients; mixed indications; no comparator
Hong et al. [18]	Clinical and instrument-based pigment improvement	Mild pain; transient erythema	No serious event reported	Manufacturer and university support	Consultant and speaker relationship	Prospective pilot	All-female cohort; nomenclature discrepancy; no comparator
Choi et al. [19]	Short-term improvement in melasma measures	None treatment-related reported	Recurrence after week 22 not assessed	No funding reported	No conflict reported	Prospective single-group	No comparator; limited durability assessment
Swali et al. [20]	Mean clearance category of 51%-75%	Expected discomfort; transient erythema	No pigmentary alteration reported	No study funding reported	Advisory-board relationship	Retrospective series	Phototype imbalance; final safety interval NR

Tattoo Removal

Within the limited 785 nm literature, the study by Bernstein et al. provides the most detailed direct tattoo dataset. This description refers to relative completeness within the 785 nm evidence base and not to high-certainty evidence. Fifteen participants with 22 professional tattoos and FSTs II-IV were enrolled, and 14 participants with 21 tattoos completed all visits. The completed tattoos contained 48 evaluable color components: black in 15 tattoos, green in 13, blue in eight, yellow in five, purple in four, and red in three [14].

Participants received four treatments at 6-10-week intervals using a 785 nm Ti:Sapphire handpiece delivering 300 ps pulses, 2-4 mm beam diameters, and fluences of 1.1-3.1 J/cm^2^. Clearance was assessed approximately eight weeks after the final treatment by blinded evaluation of standardized digital cross-polarized photographs using an 11-point scale. Mean estimated clearance was 85% for purple, 81% for blue, 74% for green, 61% for black, 11% for red, and 5% for yellow. Dispersion measures or confidence intervals for the color-specific estimates were not reported. Erythema and edema were typical, one episode of pinpoint bleeding occurred, and no scarring or pigmentary alteration was reported during the stated follow-up [14].

These percentages should therefore be interpreted as descriptive estimates from a small, single-arm, manufacturer-involved study rather than as comparative efficacy estimates. The study supports a potential niche for blue, green, and purple components, but it does not establish superiority over 532 nm, 755 nm, or 1064 nm platforms. It also does not establish safety in FSTs V-VI or durability beyond the short post-treatment interval.

Epidermal and Benign Facial Pigmentation

Chung et al. described two Asian patients with ephelides treated once with a 785 nm picosecond device at 1.2-1.3 J/cm^2^ using a 3 mm spot. The publication did not provide sufficient information in the accessible report to assign the output to a specific gain medium or to confirm the pulse duration, so these fields are recorded as not reported rather than inferred. Clinical photography showed appreciable improvement without reported complications; however, assessment was subjective, unblinded, and based on two cases, and a defined follow-up interval was not reported [15].

Hong et al. subsequently described three Korean women with FSTs III-IV and mixed facial pigment disorders treated with a HELIOS IV 785 nm Ti:Sapphire channel using fractionated and focal handpieces. The reported protocols included a 600 ps pulse duration, a 5 x 5 mm diffractive optical element (DOE) handpiece at 0.8 J/cm^2^, and a 3 mm focal handpiece at 1.2 J/cm^2^ in selected lesions. Three to four sessions were performed at four-week intervals. Photographic improvement was reported, with no PIH or depigmentation through six weeks after treatment, but the evidence remained case-level, unblinded, and heterogeneous by diagnosis [17].

A subsequent Hong et al.'s pilot study included 15 Korean women with FSTs II-IV skin who underwent five weekly dual-mode treatments. A 5 x 5 mm DOE handpiece was used at 0.8 J/cm^2^ and a 7 x 7 mm collimated handpiece at 0.3-0.4 J/cm^2^. Independent dermatologist ratings and instrument-based melanin, brown, and erythema indices showed improvement at week 8. Mild pain and transient erythema were reported without serious adverse events. Interpretation is limited by the small all-female cohort, absence of an active comparator, short follow-up, manufacturer funding, and an author industry relationship [18]. This study by Hong et al. requires explicit source terminology. Its title and methods describe a 785 nm picosecond Nd:YAG laser, whereas technical documentation for the HELIOS IV family describes a 785 nm Ti:Sapphire-generated channel pumped within an Nd:YAG-based platform. The study is therefore categorized here as HELIOS IV 785 platform evidence with a nomenclature caveat rather than being pooled uncritically as generic Nd:YAG or generic Ti:Sapphire evidence.

Melasma

Choi et al. conducted a prospective single-center, single-group before-and-after study in 18 Asian participants with melasma, including 17 women and one man with a mean age of 44.44 years. FST was not reported. Participants received 10 treatments at two-week intervals using a 785 nm Ti:Sapphire, 600 ps DOE handpiece at 0.4-0.6 J/cm^2^, a 5 x 5 mm spot, 10 Hz, and two to three passes to a mild-erythema endpoint [19].

Standardized photographs were evaluated by two independent dermatologists, and objective melanin measurements and a seven-point satisfaction scale were obtained at baseline, week 10, and week 22. Physician-rated improvement and melanin indices improved at the final assessment, and no treatment-related adverse events were reported. However, week 22 represented only approximately four weeks after completion of the 10-session course. The study did not include a control group and did not report longer-term recurrence. Accordingly, it supports only short-term, hypothesis-generating efficacy and tolerability and does not establish durable melasma control or comparative effectiveness [19].

Dermal Melanocytosis

Loh and Wu reported two nevus of Ota cases treated with neighboring Ti:Sapphire wavelengths. One patient with FST IV received three monthly 785 nm treatments at 1.0 J/cm^2^, a 4 mm spot, and four passes after limited response to prior 755 nm and 1064 nm picosecond treatment. Near-complete clearance was reported at short follow-up without pigmentary alteration. Because one case used 730 nm and one used 785 nm, the report provides proof of concept but cannot support a pooled estimate for 785 nm [16].

Swali et al. retrospectively reviewed 17 patients with brown nevus of Ota, including one FST II, one FST III, 14 FST IV, and one FST V. Treatment used a 785 nm PicoWay handpiece at 1.0 J/cm^2^ with a 4 mm spot size, three to five passes, and a dermal-edema endpoint. Patients received a mean of 3.2 sessions at two- to three-month intervals. Three board-certified dermatologists evaluated photographs with a five-point visual scale, and the mean clearance category was 51%-75% [20].

No pigmentary alteration was reported in that cohort, but the phototype distribution was heavily weighted toward FST IV, with only one patient each in FSTs II, III, and V. A uniform post-final-treatment safety-monitoring interval was not reported. Consequently, the study offers a useful preliminary signal for brown dermal melanocytosis but cannot establish safety across FSTs II-V or exclude delayed dyspigmentation, textural change, or recurrence [20].

Mechanistic Evidence

Shimojo et al. evaluated wavelength-dependent melanosome disruption using porcine retinal pigment epithelium melanosomes, dynamic light scattering, scanning electron microscopy, and Monte Carlo modeling. Experimental disruption thresholds were 0.95 J/cm^2^ for 532 nm at 375 ps, 2.25 J/cm^2^ for 730 nm at 250 ps, 2.75 J/cm^2^ for 785 nm at 300 ps, and 6.50 J/cm^2^ for 1064 nm at 450 ps. Previously reported values of 755 nm were included for contextual comparison [21].

These values are experimental thresholds under specified ex vivo conditions. They are not recommended clinical treatment fluences, maximum permissible exposures, or safety thresholds. They cannot be transferred directly to intact human skin because epidermal melanin, vascular absorption, scattering, target depth, beam diameter, and tissue heterogeneity alter delivered energy. They also cannot be applied directly to tattoo particles, whose composition and physical organization differ from melanosomes. The appropriate conclusion is limited to mechanistic plausibility for an intermediate 785 nm position, not clinical superiority or parameter guidance [21].

Neighboring 730 nm and 755 nm Evidence

The 730 nm Ti:Sapphire literature includes prospective studies of multicolored tattoos and benign pigmented lesions, while the 755 nm literature provides additional neighboring-wavelength context [22-25]. These studies support the general concept that intermediate wavelengths can target selected pigment spectra but are not direct evidence for 785 nm. Differences in absorption, pulse duration, spot size, beam profile, and device architecture preclude pooling their outcomes with 785 nm studies. Their role in this review is contextual only.

Safety and Regulatory Considerations

Higher epidermal melanin increases competition with the intended target and raises the risk of PIH, hypopigmentation, blistering, and textural change. Comparative pigment studies in Asian cohorts and multichannel picosecond reports provide useful context, but they do not establish isolated 785 nm safety in all skin phototypes [26,27]. Direct 785 nm reports include selected participants with FSTs II-V, but FST VI is absent from the direct evidence summarized above. Small samples and short or incompletely reported follow-up may fail to capture delayed dyspigmentation, textural alteration, or melasma recurrence.

Safety statements should therefore remain study-specific. Test spots, conservative fluence escalation, appropriate spot-size selection, careful endpoint monitoring, cooling when clinically indicated, adequate treatment intervals, and photoprotection remain prudent principles. The absence of an adverse event in a small series should be reported as observed tolerability during the stated follow-up rather than as proof of safety for an entire FST range.

Tattoo wavelength selection should not be based on visible color alone. Yellow pigment may respond inconsistently or poorly, and cosmetic, flesh-toned, white, or red inks containing iron oxides or titanium dioxide may undergo paradoxical darkening after laser exposure. Pigment chemistry, depth, prior treatment response, and test-spot behavior are therefore essential, particularly when the formulation is unknown [10,28]. Regulatory indications should remain clearly separated from these clinical decisions because clearance is platform- and jurisdiction-specific [12,13].

Clinical Implications and Complementary-Wavelength Framework

A practical framework should begin with the target rather than the device brand. For professional tattoos, 1064 nm remains foundational for black and deeper dark components because of its penetration and lower epidermal melanin competition. The 532 nm wavelength remains important for selected superficial red and orange components, although response varies by ink chemistry and yellow pigment may remain difficult. Intermediate wavelengths, including 785 nm, may be considered for selected blue, green, purple, and recalcitrant residual components after appropriate diagnosis and test-spot assessment.

For benign pigmentary disorders, lesion diagnosis and depth are essential. A solar lentigo, ephelis, melasma, dermal melanocytosis, and exogenous tattoo deposit should not be treated as interchangeable brown targets. The limited direct evidence suggests potential use in selected ephelides, mixed benign facial pigmentation, melasma protocols, and brown nevus of Ota, but each indication has a different biological course and recurrence risk. Melasma in particular requires conservative energy delivery, strict photoprotection, and recognition that short-term lightening does not establish durable disease control.

The clinically defensible role of 785 nm is therefore complementary: it provides an additional wavelength for selected pigment spectra or depths when established wavelengths are incomplete or less well matched to the target. Current evidence does not justify presenting 785 nm as universally superior to, or a replacement for, 532 nm or 1064 nm systems.

Limitations of the Current Evidence

The principal limitation is the absence of randomized or split-lesion head-to-head trials directly comparing 785 nm with established 532 nm, 755 nm, or 1064 nm treatment for the same target. The direct literature consists of one small prospective tattoo study, a small case series, pilot studies, a single-group melasma study, and one retrospective nevus of Ota series. Follow-up was generally short, outcome instruments were heterogeneous, and recurrence was incompletely assessed.

Device heterogeneity further limits interpretation. Studies differ in gain medium terminology, pulse duration, collimated versus DOE delivery, spot size, fluence, repetition rate, number of passes, treatment interval, clinical endpoint, photography, evaluator blinding, and outcome scale. Several reports involved manufacturer funding, manufacturer-employed authors, consultants, speakers, or advisory-board relationships. These features do not invalidate the findings but require transparent, study-specific interpretation.

This review itself also has limitations. The search used one bibliographic database, screening and extraction were performed by one author, and no validated risk-of-bias or certainty-assessment method was applied. Although the complete search strategy and record flow are reported, selective inclusion cannot be excluded. The evidence was therefore synthesized descriptively, without pooled estimates or formal comparative ranking.

## Conclusions

The 785 nm wavelength represents an emerging complementary option in tattoo and pigment treatment. The most consistent direct clinical signal concerns selected blue, green, and purple tattoo components, while evidence in ephelides, benign facial pigmentation, melasma, and brown dermal melanocytosis remains preliminary. Experimental melanosome-disruption data support mechanistic plausibility but do not define human treatment or safety thresholds.

To ensure clinically appropriate interpretation, conclusions should remain wavelength-, platform-, and indication-specific. The 1064 nm wavelength remains foundational for black and deeper pigments, 532 nm remains important for selected superficial warm-color targets, and 785 nm may expand treatment options for selected intermediate-spectrum targets. Future studies should use direct randomized or split-lesion comparisons, standardized device reporting, objective and blinded outcome assessment, longer follow-up, recurrence evaluation, and broader representation of darker skin phototypes.
